# Evaluation of the Impact of Behavioral Opportunities on Four Zoo-Housed Aardvarks (*Orycteropus afer*)

**DOI:** 10.3390/ani10081433

**Published:** 2020-08-17

**Authors:** Jennifer Hamilton, Grace Fuller, Stephanie Allard

**Affiliations:** 1 Center for Zoo and Aquarium Animal Welfare and Ethics, Detroit Zoological Society, Royal Oak, MI 48067, USA; gfuller@dzs.org; 2 National Aquarium, Baltimore, MD 21202, USA; sallard@aqua.org

**Keywords:** environmental enrichment, animal welfare, nocturnal species, goal-based enrichment, corticosterone, fecal glucocorticoid metabolites

## Abstract

**Simple Summary:**

Evaluations of enrichment are critical to determine whether or not the goals of an enrichment program are being achieved. However, evaluations can be challenging if a species’ active period occurs outside of their caretakers’ normal schedule. Aardvarks are an understudied, nocturnal species, and our aim was to assess how they used their enrichment (nightly and throughout the study), if enrichment achieved the stated behavioral goals, and the subsequent effects of goal-achieving enrichment on the aardvarks’ welfare. Four aardvarks were given seven different enrichment items based on two behavioral goals, foraging and exploring, and were observed three times during the night. The aardvarks’ enrichment use was impacted based on the goal of the enrichment, with aardvarks using the enrichment aimed at promoting foraging behavior more when enrichment was first given compared to enrichment that promoted exploring. However, exploring enrichment was used more later in the night. The enrichment did appear to achieve the stated behavioral goals, and the aardvarks continued using the enrichment throughout the entire eight-week study. In addition, there were increased affiliative and decreased agonistic interactions with more enrichment use, linking the achievement of these goals to positive indicators of welfare. The data presented contribute to the current knowledge on goal-based enrichment and its impact on animal welfare.

**Abstract:**

Evaluations of enrichment are critical to determine if an enrichment program is meeting stated goals. However, nocturnal species can present a challenge if their active periods do not align with caretakers’ schedules. To evaluate enrichment for four aardvarks housed with a natural light cycle, we provided seven different enrichment items aimed at fulfilling two behavioral goals: exploring and foraging. We wanted to understand how the aardvarks used enrichment, if enrichment promoted the defined goals, and how enrichment that achieves its goals affects welfare indicators, including rates of pacing and social behaviors, behavioral diversity, and fecal glucocorticoid metabolites. Twenty-minute observations from video were performed three times a night for a total of 224 observed hours. We found significant differences in how the aardvarks used items from the two enrichment goals throughout the night, with foraging enrichment used more than exploring at first and exploring enrichment used more later. We found that items promoted their defined goals, and aardvarks showed no evidence of habituation throughout the eight-week study. The impact on selected welfare indicators provided evidence of potentially positive changes, including increased affiliative and decreased agonistic interactions accompanying increases in goal behaviors. These results contribute to the current knowledge available on the impact of goal-directed behavioral opportunities on zoo animal welfare.

## 1. Introduction

Animal welfare is defined as the collective physical, mental, and emotional states of an animal over time [1]. Each individual experiences different welfare depending on their natural and individual history, personality, and environment [2]. Methods to assess animal welfare consist of behavioral measures, including specific behaviors such as stereotypies or social behaviors, (see [2] for review) or aggregate measures like behavioral diversity [3]. Physiological indicators can also be used to measure welfare, with glucocorticoids being the most commonly employed in zoo studies [4]. Integrated measures of glucocorticoid activity, such as fecal glucocorticoid metabolites (FGM), can summarize activity of the hypothalamic–pituitary–adrenal (HPA) axis over a period of time (usually 24–48 h for fecal samples). Longitudinal profiles of individual adrenal activity can then be compared as the animal experiences different husbandry conditions as an indication of how those conditions affect animal welfare. However, it should be noted that adrenal activity can sometimes increase during positive experiences, as well as in response to environmental threats, so careful analysis of individual hormone profiles in relation to behavioral data is needed to interpret results. Social interactions [5] and rates of abnormal behaviors, such as pacing [6], have also been linked to changes in adrenal activity and are considered indicators of animal welfare in their own right; for that reason, these factors should also be taken into account when analyzing FGM profiles. These complexities are why multiple indicators are preferable when examining the impact of an event on the welfare of an animal. One type of husbandry event that is commonly evaluated using these types of welfare indicators is the provisioning of environmental enrichment.

Increased attention on environmental enrichment is part of a broader shift towards emphasizing animal welfare in zoos and aquariums. Enrichment has been defined as “a process for improving or enhancing zoo animal environments and care within the context of their inhabitants’ behavioral biology and natural history” [7]. In the past, enrichment was mainly item based and focused on five to eight broad categories (e.g., feeding, tactile, olfactory) with the goal of providing enrichment from every category [8,9]. Although an item-based system of enrichment provides animals with events of varying degrees of stimulation, there can be a lack of understanding as to whether these events are functionally significant to the animal in question [10,11]. Mellen and MacPhee [12] proposed that zoos and aquariums should instead develop goal-based enrichment frameworks that promote species-appropriate behaviors and mitigate undesirable behaviors with the objective of improving an animal’s welfare. 

As part of this transition towards a goal-driven framework, many zoo professionals have argued that enrichment cannot be considered “enriching” based on preconceived notions alone; evaluation is needed to assess whether the enrichment is meeting an animal’s needs [11,13]. Many enrichment evaluations aim to assess differences in goal behaviors or activity budgets compared to a baseline. These evaluations can range from keepers providing quick, sometimes more anecdotal, assessments to more formal observational research. In addition, data collected on enrichment use over multiple sessions, such as habituation data, can help observers determine motivation to perform specific goal behaviors [14]. 

Enrichment evaluations are needed for all individuals due to differences between species, but also individual histories and preferences. A specific challenge of evaluating enrichment for nocturnal mammals is that the animals are typically beginning to wake as care staff are finishing their workday, so in-person enrichment evaluations are not always possible. Although informal evaluations are frequently completed, these evaluations can overlook individual behavioral changes that would indicate if the enrichment is meeting the intended goal or has positively affected animal welfare. For example, evaluations might be based on indirect evidence of use (such as food depletion or object location), but it may not be possible to link these changes to individuals if animals are housed socially. In some cases, the animal’s light cycles are shifted so that their active period corresponds with zoo operating hours, which allows for direct evaluation. Enrichment evaluations have been performed under these conditions for some nocturnal species, including kinkajous (*Potos flavus*; [15]), armadillos (*Dasypus novemcinctus*), Senegal bushbabies (*Galago senegalensis*) and two-toed sloths (*Choloepus didactylus*; [16]), and ghost bats (*Macroderma gigas*) and yellow-bellied gliders (*Petaurus australis*; [17]). However, the welfare implications of modifying light cycles and illuminating the habitat during the dark phase for ease of visitor viewing are complex and not fully understood [18]. Another option is to use technology to perform observations. Low-light or infrared camera systems are an option that permit observers to watch an animal’s nocturnal activity while having minimal impact on the animal. 

Due to their nocturnal and semi-fossorial nature, research on the natural history of aardvarks (*Orycteropus afer*) has been scarce. In the wild, aardvarks have been tracked traveling 1–2 km a night [19] to find food, burrow sites, and mates [20,21]. Although predated items vary throughout the year, an aardvark can have up to 200 foraging bouts per night [21]. There is also little research available on aardvark welfare in captivity. Allard, Fuller, and Hamilton [22] used infrared cameras to assess activity budgets and collected fecal samples for hormone analysis to compare the welfare of aardvarks over time and in different habitat spaces at the Detroit Zoo. The data showed that FGM concentrations were lower when the aardvarks spent more time exploring their habitat [22], suggesting that enrichment designed to encourage investigation may be a way to positively influence the welfare of aardvarks in captivity. Based on our previous research and aardvark natural feeding and traveling behaviors, we therefore chose to focus this study on the behavioral goals of increasing rates of foraging and exploring the environment. This exploratory study addressed two primary issues: the extent to which different types of enrichment promote their intended behavioral goals and their subsequent effects on indicators of animal welfare. We asked the following specific questions:

(1) How did the amount and rate of use vary depending on the stated enrichment goal?
(2) Which enrichment items better achieved their stated goals based on use throughout the night? What about throughout the study (i.e., was habituation observed)?
(3) Following this logic, we developed the following question post hoc. Which enrichment items better achieved their stated goal based on promoting overall rates of goal behaviors, including behaviors directed towards non-enrichment as well as enrichment items?
(4) Did certain enrichment types or increased enrichment use correspond to positive changes in specific welfare indicators, including FGM concentrations, behavioral diversity, pacing, or social behaviors?

Based on these questions, we explored how meeting behavioral goals translated into measurable improvements in welfare for the aardvarks in this study.

## 2. Materials and Methods 

This study was reviewed and approved by the Senior Leadership in Animal Welfare and Management Committee of the Detroit Zoological Society (DZS).

### 2.1. Subjects and Housing

Four aardvarks housed in two social groups at the Detroit Zoo in Royal Oak, MI, USA, participated in this study. Although a small sample size, this represents 11.4% of the aardvark population housed in AZA facilities [23]. A fourteen-year-old male, Baji, and a six-year-old female, Roxaane, comprised one group. A second group was comprised of a thirteen-year-old female, Rachaael, and a three-year-old female, Kaatie. Baji and Rachaael are the parents of both younger aardvarks. During the study, the aardvarks rotated daily between two behind-the-scenes indoor habitats of similar size with varied substrates and features (Table 1). The habitats shared a barrier that allowed for limited contact between the two groups. Light cycles for both habitats were created using a combination of artificial lighting and skylights, with the dark phase lasting from approximately 22:00 to 08:00 during this study. These aardvarks have never been housed on a reversed light cycle at the Detroit Zoo due to concerns about how that practice could potentially affect their welfare. Animal care staff usually serviced the habitat between 16:00 and 18:00, while the aardvarks slept. Fresh food (roughly 0.68 kg per aardvark of Mazuri^®^ insectivore diet; St. Louis, MO, USA) and water were provided nightly by 22:00, prior to the final light being turned off and animal care staff leaving the building. 

### 2.2. Enrichment Events

We chose seven enrichment items to encourage two main behavioral goals: foraging and exploring the environment (Table 2). These items were all previously approved through the DZS enrichment approval process and were not novel to the aardvarks. Enrichment items usually were distributed between 21:00 and 22:00 each night. Each item was assigned to a day of the week to create a recurring schedule that was repeated for eight weeks, allowing each aardvark to receive each item in each habitat four times. As the aardvarks were accustomed to receiving enrichment daily, and we did not want to impact their welfare negatively by removing a potentially positive event, we did not include a ‘no enrichment’ condition. No enrichment additional to the study items was given to the aardvarks during this time period.

Paper towel treat tubes, slow feeders, and rubber toys filled with food were all chosen to decelerate consumption of food, because the animal had to manipulate the device with their tongue, nose, and front feet to ingest the food. Slow feeders consisted of commercially available bowls with plastic obstructions. Cricket containers provided the aardvarks with the opportunity to manipulate an object to gain access to live prey in a semi-naturalistic manner. We chose the warthog (*Phacochoerus africanus*) scent, as we thought this scent may be relevant to the aardvarks, as previously, warthogs were housed in the same building as the aardvarks, and warthogs and aardvarks share similar geographical ranges in the wild [24]. We chose to include perfume and Boomer balls^®^ (Grayslake, IL, USA) as exploring enrichment because they were commonly used enrichment for the aardvarks. The last exploring enrichment, a pool with water, was chosen because the female pair of aardvarks were observed playing in their water bowl and wading through shallow water when offered. We thought access to a larger body of water might increase locomotion and investigation near the pool. Aardvarks were provided with at least three identical items each day to avoid competition for enrichment items, with one exception. The pool was large enough that one aardvark could not monopolize the use of the pool, so only a single pool was provided. The number of enrichment items was consistent from week to week, and the amount of food provided was consistent among the foraging days.

### 2.3. Behavioral Data Collection

We collected data from a prerecorded video from 15 December 2017 to 9 February 2018. The video was recorded using XProtect^®^ Smart Client 2017 software (Brøndby, Denmark). Six observers were trained to collect data, and all demonstrated >90% inter-observer reliability based on the percent difference in behaviors scored during three video observations. Inter-observer reliability was rechecked every three months during the video-coding process.

Video observations consisted of instantaneous scan sampling at one-minute intervals, all-occurrence, and continuous sampling. Three 20-min observations on each aardvark occurred seven days a week for eight weeks, resulting in a total of 168 observations or 56 h for each aardvark. The first observation of the night started when the animal care staff set down the last enrichment item in the aardvarks’ habitat, usually between 21:00 and 22:00 (PM). The second and third observations of the night took place between 00:30 and 01:30 (MID), and 04:00 and 05:00 (AM). At each scan, we recorded the focal’s behavior (Table 3), location within the habitat, proximity to enrichment (contact, within one body length, or greater than one body length), and substrate (dirt, cement, rubber mats, shavings, plastic culvert, nest box, water, or unclear). 

All-occurrence sampling was used to record affiliative and agonistic interactions, and continuous sampling was used to record all pacing and investigating enrichment events. At least five seconds separated all-occurrence or continuous events of the same behavior.

### 2.4. Hormone Sampling

Fecal samples were collected once a day in the evening (Baji: 26 samples; Roxaane: 37 samples; Rachaael: 27 samples; Kaatie: 35 samples). Animal care staff collected fecal samples within three hours of defecation. As they were housed in pairs, aardvarks received different colored food paste in avocado to make their feces distinguishable [25]. Fecals were collected after care staff cleaned the habitat to eliminate the risk of collecting older samples. Samples were immediately stored at −20 °C. 

Fecal hormone metabolites were extracted and analyzed using methods reported in Allard et al. [22]. Briefly, samples were lyophilized and sifted, and then, 0.2 ± 0.01 g fecal powder was extracted in 2.0 mL, 90% ethanol by shaking, centrifuging, and evaporating the supernatant under forced air at 35 °C. Samples were reconstituted in assay buffer, and FGM concentrations were measured using a commercial assay kit for corticosterone (#K014-H1, Arbor Assays, Ann Arbor MI) that was previously validated chemically and biologically for these aardvarks at the Detroit Zoo [22]. All samples were analyzed in duplicate, and samples with a coefficient of variation (CV) greater than 15% were reanalyzed. The average intra-assay CV was 1.76%. Internal controls were analyzed on each plate, and the average inter-assay CV was 2.82%. Based on the previous validation results [22], dates for FGM concentrations were adjusted backward by 24 h to account for lag time to hormone excretion when making comparisons to behavioral data.

### 2.5. Statistical Analyses

We summarized percentages of scan variables and rates from all-occurrence variables by observation and by night for each individual. Continuous variables were also summarized as percentages using durations and as rates using counts. Data were analyzed without controlling for visibility, because both the focal aardvark and the enrichment were out of view less than one percent of the time. Although we recorded information on location within habitat and proximity to enrichment and substrate, because these variables did not answer our main questions, they will not be addressed further. Goal achievement was assessed by looking at three specific behaviors: total time foraging (scans of foraging and investigating foraging enrichment combined), total investigating non-food items (scans of investigating and investigating exploring enrichment combined), and locomotion. Although total time foraging and total time investigating non-food items have a clear connection to the goals of the enrichment items (i.e., foraging and exploring), we included locomotion as an indication of the efficacy of exploring enrichment, as movement throughout the habitat would present more opportunities for investigation. Behavioral diversity (H) was calculated each night from scan data using the Shannon–Wiener Diversity Index [26]. Data were visualized using Microsoft Excel 2016 (Redmond, WA, USA). 

We analyzed the outcome variables using generalized linear mixed models (GLMM) in SPSS v. 25 (IBM Corporation, Armonk, NY, USA). In the past, the aardvarks’ behavioral patterns varied in different habitats [22]. To control for this variation, we used habitat as part of a random slope term that also included individual identity and either the seven specific enrichment items or the two stated goals of the enrichment (i.e., foraging or exploring). We then modeled each target variable twice to match the corresponding slope: once including fixed factors for enrichment item and once including fixed factors for stated goal. All model building was conducted in a top-down manner, removing non-significant fixed factors, and re-running the model until only significant fixed factors remained. However, we always retained either enrichment goal or specific enrichment item as fixed factors in the models because they pertained to our main study questions. We calculated degrees of freedom for all models using a Satterthwaite approximation. 

We compared time spent using enrichment (counts of scans from each observation) and rates of enrichment use (counts of continuous events from each observation) using GLMMs with negative binomial distributions and log link functions. In these models, we included the following fixed factors: enrichment (stated goal or specific item), categorical time of night (PM, MID, or AM), and an interaction term for enrichment (stated goal or specific item) × time. These models used a variance components covariance structure and a random slope term matching the specific enrichment or stated goal of enrichment as described above.

To examine habituation, we modeled use of the enrichment over the eight-week study period using data aggregated by night rather than individual observations. For these GLMM, we used time in the study (two-week period from beginning of study; four periods in total) as a categorical variable, because the change in enrichment use from week to week did not follow a consistent, linear pattern. We used four two-week periods so that each aardvark had received all the enrichment once in each of the two habitats as an additional control for the impact of habitat on enrichment use. For fixed factors in these models, we used categorical time in the study, enrichment goal or specific enrichment item and the interaction of enrichment and time. We used the same random slope terms previously described and a variance components covariance structure. 

To assess the extent to which enrichment items achieved the overall goals, we modeled total time foraging, total time investigating, or locomotion as target variables. For these models, we used data aggregated by night in GLMM with negative binomial distributions and log link functions. We used two-week period of the study (four total periods) as a continuous predictor and enrichment (specific or stated goal) as fixed factors, but we were unable to fit models with terms for interactions between time and enrichment. These models used the same random slopes previously described. Models for foraging and investigating included a variance components covariance structure, while the model for locomotion used an AR1 covariance structure. 

Next, we modeled the effects of enrichment on welfare indicators using data aggregated by night. For these models, we used time (two-week period of the study) as a continuous predictor. We modeled log-transformed FGM concentrations using a normal distribution and identity link function. These models included the aforementioned random slope terms and a variance components covariance structure. To assess how FGM concentrations varied in response to enrichment characteristics, we included the following fixed factors: continuous time, enrichment (stated goal or specific item), total time of enrichment use (seconds), and counts of enrichment events. To explore alternative explanations for FGM variations, we also included the following fixed factors in models: sample collection time, total time pacing (seconds), counts of pacing events, and counts of total agonistic and affiliative interactions. These models would not support an interaction between time and enrichment. We also modeled behavioral diversity (H) using a normal distribution and identity link function, with the same random slope terms; however, this model used an AR1 covariance structure. Fixed factors for behavioral diversity included time and enrichment (specific or goal); we did not use behaviors as predictors in the behavioral diversity models because those behaviors were used to calculate H scores. 

Finally, we assessed how enrichment characteristics affected levels of abnormal and social behaviors. We modeled pacing and initiating affiliative interactions using Poisson distributions and log link functions, and we modeled initiating agonistic interactions using a negative binomial distribution and a log link function. We were unable to fit models for pacing, initiating affiliative interactions, or initiating agonistic interactions using the specific enrichment (each of the seven items) due to the relative rarity of these behaviors. Therefore, fixed factors for these three behaviors included: time in the study (continuous two-week period), stated goal of enrichment, counts of enrichment events, and total time in enrichment events. We also included the interaction between the stated goal and the count of enrichment events, and the interaction between the stated goal and the total time in enrichment events, as fixed factors in the pacing model. These models used variance components covariance structures and the aforementioned random slope terms, with the exception of the model for initiating affiliative interactions, which would only support a random intercept. 

In the results, we present b estimates (or exp(b) for count variables) along with the 95% CI for continuous predictors. For categorical predictors, we made pairwise comparisons using estimated marginal means and adjusted for multiple comparisons using the least significant difference. Standard errors are based on *n* = 4 aardvarks unless otherwise indicated. We considered *p* < 0.05 to indicate statistically significant results. Due to the large number of pairwise comparisons, we only present values for statistically significant results for comparisons that we thought answered the project questions. Please see the supplemental materials for all other pairwise comparisons. 

## 3. Results

### 3.1. Enrichment Use: Time of Night

**Figure 1:**
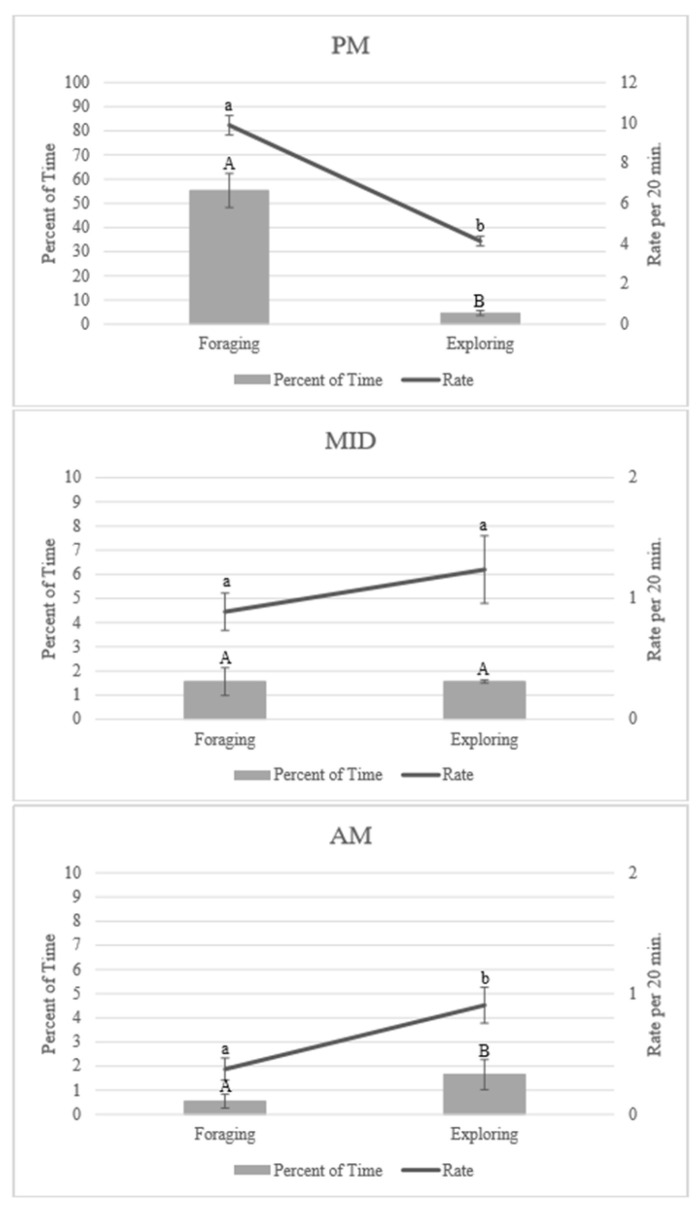
Percent of time spent using enrichment and the rate of enrichment use for each stated enrichment goal by the time of night (mean ± SE; *n* = 4). PM observations occurred between 21:00 and 22:00, MID observations occurred between 00:30 and 01:30, and AM observations occurred between 04:00 and 05:00. Significant pairwise comparisons (*p* < 0.05) within each timeframe are noted on the graph with different letters, using uppercase letters for percent of time and lowercase letters for rate per 20 min. Note that the scale for enrichment use changes between the PM, MID, and AM graphs. Pairwise comparisons can be found in Table S2.

**Figure 2:**
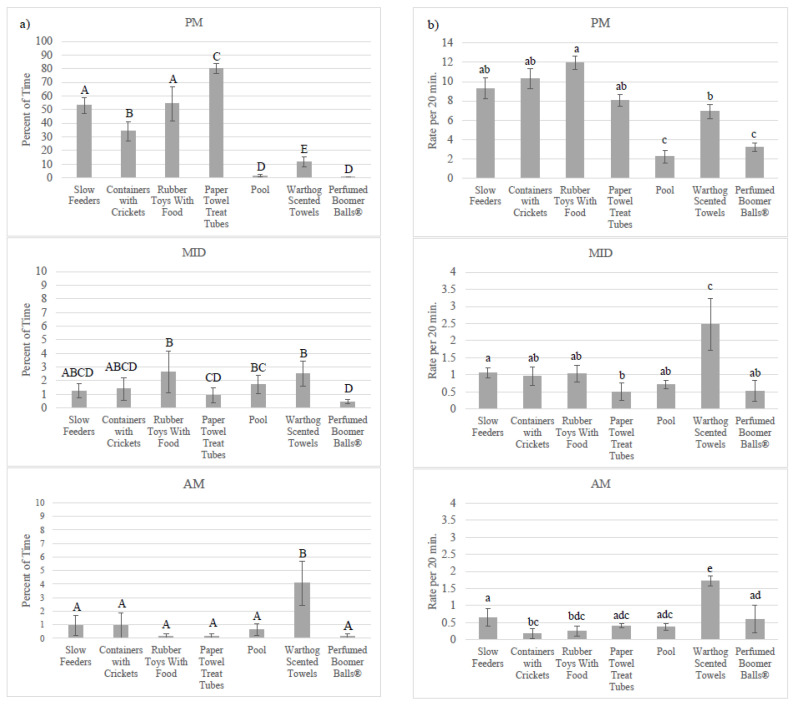
Percent of time spent using enrichment (**a**) and the rate of enrichment use (**b**) for each enrichment item by the time of night (mean ± SE; *n* = 4). PM observations occurred between 21:00 and 22:00, MID observations occurred between 00:30 and 01:30, and AM observations occurred between 04:00 and 05:00. Significant pairwise comparisons (*p* < 0.05) within each timeframe are noted on the graph with different letters, using uppercase letters for percent of time and lowercase letters for rate per 20 min. Note that the scale for enrichment use changes between the PM, MID, and AM graphs. Pairwise comparisons can be found in Table S4.

Based on scan data, the aardvarks spent significantly more time investigating the foraging enrichment (mean ± SE: 19.14 ± 2.24% of time) compared to the exploring enrichment (2.60 ± 0.31% of time; Figure S1), but patterns of enrichment use were significantly influenced by time of night (F_2,664_ = 85.22, *p* < 0.001; Figure 1; Tables S1 and S2). Event counts for using foraging and exploring enrichment also had a significant interaction between stated enrichment goal and time of night (F_2,664_ = 32.30, *p* < 0.001). Use of items from both stated enrichment goals decreased throughout the night. Foraging enrichment was used the most during PM observations (55.31 ± 7.08% of time) compared to exploring enrichment (4.58 ± 1.02% of time). During MID observations, use of enrichment was similar between the two enrichment goals (foraging: 1.56 ± 0.57% of time; exploring: 1.56 ± 0.06% of time), but during AM observations, exploring (1.65 ± 0.63% of time) was used more than the foraging (0.55 ± 0.29% of time) enrichment. Rates of enrichment use followed a similar pattern. Time spent using and event counts for using the individual items also varied significantly among the three time periods (F_12,649_ = 14.59, *p* < 0.001 and F_12,649_ = 7.32, *p* < 0.001, respectively; Figure 2; Tables S3 and S4). 

### 3.2. Enrichment Use: Goal Achievement

**Figure 3:**
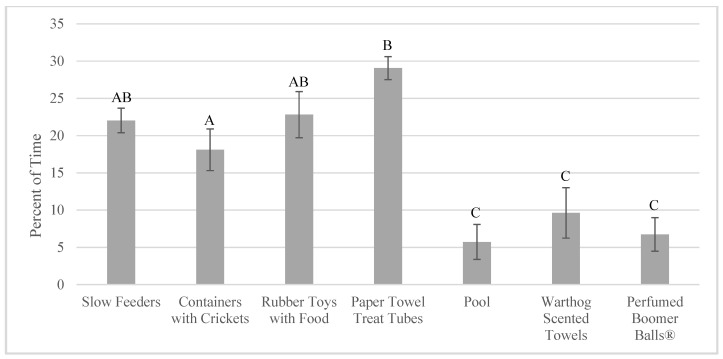
Percent of total time foraging when each enrichment item was available (mean ± SE; *n* = 4). Significant pairwise comparisons (*p* < 0.05) are noted on the graph with different uppercase letters. Pairwise comparisons can be found in Table S5.

Total time foraging (for enrichment and non-enrichment items) was higher when foraging enrichment was given (23.00 ± 2.11% of time) compared to when exploring enrichment was given (7.37 ± 2.64% of time; F_1,11_ = 48.37, *p* < 0.001). There were significant differences in total time foraging based on comparisons of specific enrichment items as well (F_6,38_ = 23.53, *p* < 0.001; Figure 3; Table S5).

**Figure 4:**
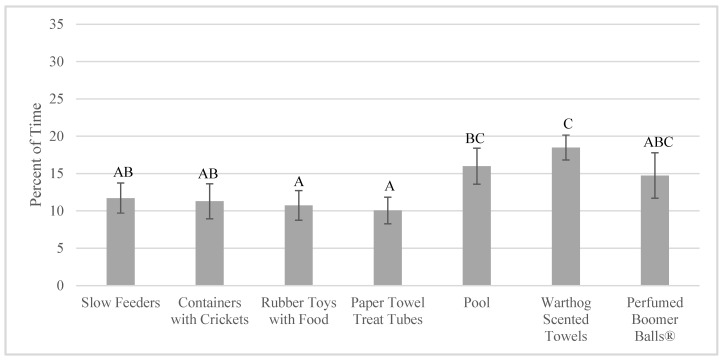
Percent of total time investigating non-food items when each enrichment item was available (mean ± SE; *n* = 4). Significant pairwise comparisons (*p* < 0.05) are noted on the graph with different uppercase letters. Pairwise comparisons can be found in Table S6.

Conversely, total percent of time investigating non-food items (enrichment and non-enrichment combined) was lower when foraging enrichment was given (10.95 ± 1.54% of time) compared to when exploring enrichment was given (16.41 ± 2.22% of time; F_1,12_ = 14.475, *p* = 0.002). There was a significant difference in total time investigating non-food items based on which enrichment item was given (F_6,45_ = 3.143, *p* = 0.012; Figure 4; Table S6). 

**Figure 5:**
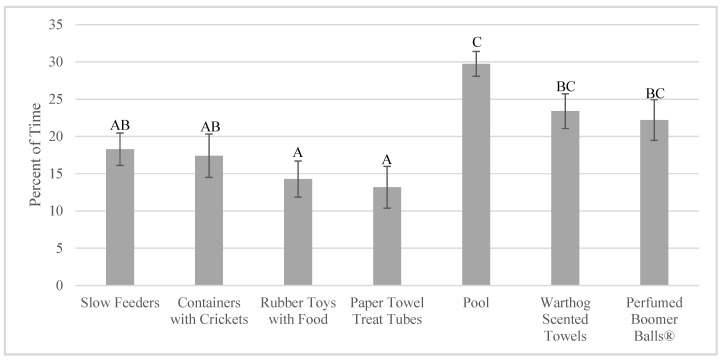
Percent of time locomoting when each enrichment item was available (mean ± SE; *n* = 4). Significant pairwise comparisons (*p* < 0.05) are noted on the graph with different uppercase letters. Pairwise comparisons can be found in Table S7.

Finally, the aardvarks overall locomoted more when exploring enrichment was given (25.10 ± 1.56% of time) compared to when foraging enrichment was given (15.78 ± 1.89% of time; F_1,222_ = 22.16, *p* < 0.001). In addition, there was a significant difference in time locomoting based on which specific enrichment item was given (F_6,46_ = 3.55, *p* = 0.006; Figure 5; Table S7).

### 3.3. Enrichment Use and Goal Achievement: Change over Time (Habituation)

**Figure 6:**
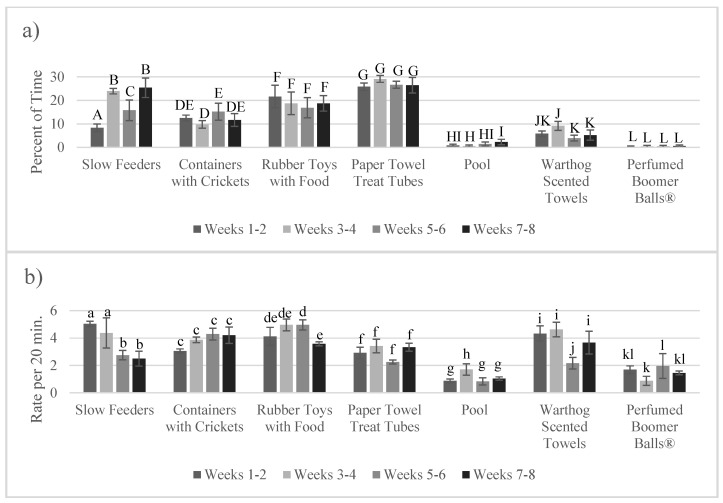
Depiction of percent of time (**a**) and rate (**b**) of using each enrichment item throughout the study (mean ± SE; *n* = 4). Significant pairwise comparisons (*p* < 0.05) within each enrichment item are noted on the graph with different letters, using uppercase letters for percent of time and lowercase letters for rate per 20 min. Pairwise comparisons between enrichment items are not shown. Pairwise comparisons can be found in Table S9.

The percent of time using enrichment over the length of the study (in two-week periods) did not differ overall (F_3,216_ = 1.875, *p* = 0.135) or as an interaction (F_3,216_ = 0.67, *p* = 0.574; Table S8) with the stated enrichment goal (i.e., foraging and exploring). Counts of enrichment use events over the length of the study (in two-week periods) did not differ overall (F_3,219_ = 1.787, *p* = 0.150) or as an interaction (F_3,216_ = 0.61, *p* = 0.608; Table S9) with the stated enrichment goal. However, there was a significant interaction for specific enrichment items and time in the study (time spent using: F_18,196_ = 3.58, *p* < 0.001; count of events: F_18,196_ = 2.92, *p* < 0.001; Figure 6). Although the aardvarks varied how much they used the enrichment between the two-week periods, only one item showed significant changes in use when the first two weeks of the study were compared with the last two weeks of the study. Slow feeders were used for a higher percentage of the time during weeks 7–8 compared to weeks 1–2 (t_35_ = −4.93, *p* < 0.001), but counts of events using slow feeders were lower in weeks 7–8 compared to weeks 1–2 (t_196_ = 3.29, *p* = 0.001). 

We also examined whether there were changes in achieving goal behaviors over the course of the study, which would suggest habituation effects. Total time foraging from all food items did not differ based on the length of time in the study for the stated enrichment goal (F_1,221_ = 1.07, *p* = 0.303) or the specific enrichment items (F_1,216_ = 1.59, *p* = 0.209). Total time investigating non-food items did not differ based on the length of time in the study based on the stated enrichment goal (F_1,221_ = 0.096, *p* = 0.757) or the specific enrichment items (F_1,216_ = 0.047, *p* = 0.829). Finally, time locomoting did not differ based on the length of time in the study based on the stated enrichment goal (F_1,221_ = 0.75, *p* = 0.386) or the specific enrichment items (F_1,216_ = 0.87, *p* = 0.351).

### 3.4. Effects on Indicators of Welfare

FGM concentrations averaged 20.00 ± 1.84 ng/g when foraging enrichment was present and 19.34 ± 1.69 ng/g when exploring enrichment was present. Overall, FGM concentrations were not significantly related to the stated enrichment goal (F_1,105_ = 0.48, *p* = 0.49) or enrichment item (F_4,94_ = 1.51, *p* = 0.21; Table S10). Values for the remaining fixed factors are based on models using the stated enrichment goal and were non-significant for: time in the study (F_1,98_ = 0.05, *p* = 0.81), sample collection time (F_1,104_ = 0.28, *p* = 0.60), the interaction of stated goal and samplecollection time (F_1,105_ = 0.43, *p* = 0.51), amount of time spent using enrichment in seconds (F_1,103_ = 0.07, *p* = 0.79), amount of time spent pacing in seconds (F_1,101_ = 0.09, *p* = 0.76), count of enrichment events (F_1,99_ = 0.21, *p* = 0.65), count of pacing events (F_1,103_ = 0.004, *p* = 0.95), total affiliative interactions (F_1,99_ = 0.05, *p* = 0.81), or total agonistic interactions (F_1,106_ = 0.74, *p* = 0.39).

All enrichment had a nightly average behavioral diversity (H) of 1.60 ± 0.04 (*n* = 7), and the range of average behavioral diversity scores for each item ranged from 1.51 to 1.71. Behavioral diversity was not related to the stated enrichment goal (F_1,174_ = 0.005, *p* = 0.943) but was related to individual enrichment items (F_6,207_ = 2.61, *p* = 0.019; Table S11). Although most pairwise comparisons for behavioral diversity between the items were not significant and are not reported here, there were a few significant pairwise comparisons. Perfumed Boomer balls^®^ had lower nightly behavioral diversity than warthog scented towels (t_207_ = −3.03, *p* = 0.003) and cricket containers (t_207_ = −2.56, *p* = 0.011). Warthog scented towels had higher behavioral diversity than pools (t_207_ = 1.99, *p* = 0.048), paper towel treat tubes (t_207_ = 1.99, *p* = 0.048), and rubber toys with food (t_207_ = 2.90, *p* = 0.004). Containers with crickets were associated with higher behavioral diversity than rubber toys with food (t_207_ = 2.43, *p* = 0.016). Finally, there was a significant decrease in behavioral diversity over the study period (F_1,193_ = 5.89, *p* = 0.016, *b* = −0.039, 95% CI [−0.071, −0.007]).

Although counts of pacing events were slightly higher for exploring enrichment (0.18 ± 0.11 events per 20 min) compared to foraging (0.11 ± 0.07 events per 20 min), the count of pacing events was not significantly predicted by the stated enrichment goal (F_1,13_ = 0.02, *p* = 0.897) or the total time spent on enrichment events (F_1,221_ = 0.89, *p* = 0.345).

Counts of initiating affiliative interactions were not significantly related to the time in the study (F_1,219_ = 0.03, *p* = 0.857) or the total amount of time an aardvark used enrichment (F_1,219_ = 0.01, *p* = 0.92). However, the stated goal of the enrichment provided significantly influenced the counts of affiliative interactions initiated, with 0.30 ± 0.09 affiliative interactions per 20 min with exploring enrichment and 0.18 ± 0.04 affiliative interactions per 20 min with foraging enrichment (F_1,221_ = 12.57, *p* < 0.001). There was also an overall effect that with higher counts of enrichment events, the higher the level of affiliative interactions (F_1,221_ = 11.68, *p* = 0.001, exp(*b*) = 1.06, 95% CI [1.02, 1.10]). The initiation of agonistic interactions was not related to the stated goal of the enrichment (F_1,45_ = 1.22, *p* = 0.27), time in the study (F_1,220_ = 2.47, *p* = 0.12), or count of enrichment events (F_1,219_ = 1.11, *p* = 0.29). However, total time engaged with enrichment was related to initiating agonistic interactions (F_1,220_ = 7.10, *p* = 0.01, exp(*b*) = 0.99, 95% CI [0.99, 1.00]). The more time that aardvarks spent with enrichment, the fewer agonistic interactions were observed, although the effect was relatively minor based on the coefficient estimates.

## 4. Discussion

### 4.1. Enrichment Use throughout the Night

The aardvarks spent more time investigating foraging enrichment compared to exploring enrichment, in addition to engaging in more foraging bouts. High rates of foraging enrichment use for short periods of time reflect a natural pattern of foraging for aardvarks. In one night, wild aardvarks can have up to 200 foraging bouts, rarely lasting longer than two minutes per bout [21]. Food is a primary reinforcer for animals, and foraging enrichment should continue to be reinforcing until the food is depleted. Thus, designing enrichment opportunities that allow for repeated use over longer periods of time promotes a species-typical foraging pattern for aardvarks. Enrichment geared toward habitat exploration may have been less reinforcing as the habitat spaces were well known. Nonetheless, just because enrichment aimed at exploring the environment was used for shorter amounts of time does not mean the goal of this enrichment is any less valuable. Additionally, it is important to note that based on the current data, we cannot dismiss the possibility that exploring enrichment was used less than foraging enrichment because the items chosen to promote exploration, such as the perfumed Boomer balls^®^, were less meaningful to the aardvarks.

There was, however, some evidence to suggest that the aardvarks did find some of the exploring enrichment rewarding, as shown by the continued use of these items throughout the night. The aardvarks significantly decreased their use of foraging enrichment after the evening period, presumably due to the items being depleted of food. Although we do not know if the olfactory enrichment held its scent throughout the night, the aardvarks were more likely to spend time investigating the exploring enrichment compared to foraging enrichment during the last observation period of the night (04:00–05:00). Decreases in enrichment use over an animal’s active period are expected and have been documented [27,28], but the way enrichment use changes over time based on the stated goal of the enrichment has not been well elucidated. The duration of engagement with specific enrichment items and stated enrichment goals could potentially be used to target behaviors that occur at a particular time of day or (in the case of nocturnal animals) night, when keepers are not present.

### 4.2. Achieving the Goal

To be a success, goal-based enrichment should promote a “goal” that may have an impact beyond just using the enrichment item [12]. Thus, an enrichment item that promotes a foraging goal should, in theory, result in more total foraging (enrichment-directed and other foraging behavior) on days that enrichment is given versus days it is not. Overall, both stated enrichment goals were achieved by the enrichment provided to accomplish that goal. Days when foraging enrichment was given had a higher percentage of the time spent in foraging behaviors, and days when exploring enrichment was given had a higher percentage of the time spent on investigating non-food items, as well as locomotion (taken together to describe exploration). However, some specific enrichment items did achieve their stated goals better than others. Although all foraging enrichment promoted more total foraging than exploring enrichment, paper towel treat tubes appeared to be the best at achieving this goal. Compared to other enrichment items, warthog scented towels promoted more time spent investigating non-food items, and pools promoted more time spent locomoting.

The enrichment that showed the fewest significant differences in achieving the designated goal behaviors was the perfumed Boomer balls^®^. This could be because, compared to the other enrichment items, perfumed Boomer balls^®^ were not as meaningful to the aardvarks. The two olfactory scents (warthog and perfume) included in these evaluations were presented on two different base items that both had functions in addition to holding scents. Due to the different motivations to use a Boomer ball^®^ versus a towel, it is difficult to say that the specific scent caused the differences in goal achievement. However, one study with black-footed cats (*Felis nigripes*) showed higher use of scents, a proxy value for goal achievement, that were biologically relevant compared to other scents [29]. Overall, little empirical research has been completed to test the relevance of certain scents, and further research is needed to determine which scents are biologically relevant to which species [30]. In addition, the pool included water, which is a critical resource for animals, and this may have contributed to the pools being more meaningful to the aardvarks and therefore, achieving their enrichment goal. However, we only occasionally observed the aardvarks drinking from the pool, and more research is needed to determine if the aardvarks’ interest in the pool was driven by a biological desire for water or another factor.

### 4.3. Lack of Habituation to Enrichment

The aardvarks did not show a decrease in investigating any of the seven enrichment items over the duration of the study. This lack of habituation may have been influenced by the week between presentations of the same item. Intermittent presentation of enrichment has been shown to help maintain enrichment use [27,31]. The overall lack of habituation may also provide further evidence that the enrichment was motivating for the aardvarks, and that through enrichment use, the aardvarks were reinforced to perform meaningful behaviors throughout the study.

### 4.4. Impact of Enrichment Use on Welfare Indicators

The aardvarks’ enrichment use affected some of the chosen welfare indicators, but not all. We chose four indicators to explore the welfare impacts of enrichment: FGM concentrations, behavioral diversity, pacing, and social interactions. These indicators were chosen because they have been linked to welfare in other species [2,3].

Glucocorticoids provide a non-invasive measure of an animal’s HPA activation, which can be associated with stress and negative welfare, but also with exercise and beneficial stressors like social introductions [2]. The potential for HPA activation to reflect both positive and negative factors could account for why the current study and others have shown no clear impact of enrichment on glucocorticoid concentrations. Another explanation may be that the lack of change in FGM concentrations is indicative of the aardvarks being accustomed to the environment and daily husbandry routine, including enrichment, which was essentially the same before and during the study period. Thus, the aardvarks may have experienced a floor effect in their glucocorticoid concentrations, meaning that it may have been difficult to detect any small decrease in glucocorticoids if baseline levels were already low [32]. Although a floor effect may be one explanation for the results in this study, we have no specific evidence that this is the case. When designing the study, we made a conscious decision not to include a control condition without enrichment because we felt this might negatively affect the aardvarks’ welfare. However, this lack of a control condition may have restricted the range of welfare the aardvarks experienced, as both stated enrichment goals were achieved by the items provided. This restriction may have decreased our ability to detect the impact of the stated enrichment goals on specific indicators or to identify if a floor effect occurred. We acknowledge that this is a limitation of the current study that makes it challenging to assess how the stated goal of enrichment affected the aardvarks’ FGM concentrations.

Although the aardvarks did not experience a wide range of nightly behavioral diversity, there were a few notable findings. Warthog scented towels and cricket containers were found to produce the highest behavioral diversity and perfumed Boomer balls^®^ the lowest. Behavioral diversity, which is calculated to include the breadth of behaviors displayed as well as their frequency, is thought to be an indicator of welfare, with higher levels of behavioral diversity equating to positive welfare [3]. The variation in behavioral diversity seen here may indicate that some enrichment items, but not stated goals of enrichment, had a stronger impact on the aardvarks’ welfare or were more functionally meaningful than others. However, our findings also highlight some of the potential drawbacks of behavioral diversity as a welfare indicator that Cronin and Ross [33] have previously reported. Enrichment use and behavioral diversity were not independent of each other, as investigating enrichment was a behavior recorded. In addition, the calculation of behavioral diversity in this study relied on broad behavior categories, which were necessitated by the difficulty in discerning detailed behaviors using our video system, but could have diminished the behavioral diversity results. Finally, providing enrichment to promote a specific goal behavior may have caused an increase in one specific behavior instead of increasing the variety of behaviors. This could have caused lower behavioral diversity results, and if that was the case, lower behavioral diversity may not be indicative of an individual’s welfare. Given these limitations of behavioral diversity as a welfare indicator, the reason for a decrease in behavioral diversity over the course of the study is unclear.

Enrichment has been shown to successfully decrease stereotypic behaviors in some situations but not all [34], including the current study. In general, the interpretation of stereotypic behavior, such as pacing, is that it is a negative indicator of welfare; although, the implications of current versus past welfare, and coping versus frustration behavior, are less clear at this time [2]. The motivation the aardvarks felt to perform the pacing behavior is also not clear at this time. Mason et al. [34] hypothesized that if enrichment does not target the underlying motivation to perform a stereotypic behavior, it may have less of an impact. Additionally, the relatively low rates of pacing behavior displayed by the aardvarks in the current study (coupled with the lack of a control condition) could also explain why we did not see effects of enrichment on this measure, similar to the potential “floor effect” observed in relation to glucocorticoids. Aardvarks showing higher rates of stereotypic behavior may be more affected by enrichment use, or perhaps, the foraging and exploring enrichment impacted pacing equally.

Finally, a decrease in agonistic behaviors and an increase in affiliative behaviors related to enrichment use, as seen in the current study, could reduce stress related to social encounters and be beneficial for an individual’s welfare. Whitham and Wielebnowski [2] discuss how affiliative behaviors could be a sign of positive welfare in social species. However, this may also be true in socially housed species that would naturally be solitary. Conversely, eight weeks was a significant amount of time, and changes in social interactions within a group could be related to seasonal behavior or sex hormone changes in those individuals that may not have been identified by our methods, especially as we did not see any significant differences in FGM concentrations. Although more research is needed, if different enrichment goals impact the opportunity for social encounters as suggested by the current study, then the behavioral goal of enrichment given during introductions and other socially sensitive times should be considered carefully.

## 5. Conclusions

We recognize that four individuals is a small sample size, even though we were able to perform inferential statistics, and caution should still be taken when making inferences about the broader population of aardvarks in zoos due to potential individual and sex differences. For example, Baji, the male aardvark, used the warthog scented towels almost twice as much as the three females. Baji’s response could have been due to sex differences that a small sample size made difficult to explore, or his response could be due to individual preferences. Regardless of the reason, it would be advisable to use caution when applying these results to other aardvarks. Ultimately, welfare and preferences are both individual concepts, and what is successful for one individual or group may not hold for another [35,36].

For this study, we explored the success of enrichment for a group of captive aardvarks using a hypothetical and goal-driven framework. We argue that enrichment should be developed with the aim of promoting specific goal behaviors appropriate to the species and individual in question, and that evaluating whether these goals are achieved is the only way to determine if an event is genuinely enriching an individual’s life. Difficulties can arise when a species’ active period does not coincide with the workday of animal care staff; however, an animal’s welfare state fluctuates over time, so it is essential to assess events that could impact their welfare across the 24 h in a day, 7 days a week [37]. The data collected outside of the normal workday can cultivate an understanding of how to provide better welfare in a captive environment and may be particularly critical for nocturnal species. This study is a first step in collecting more information on an understudied nocturnal mammal and the enrichment that can impact their night-to-night welfare.

## Figures and Tables

**Table 1:** Description of indoor habitats.

Indoor Habitat	Features
Upper Habitat	Cement habitat with two wooden nest boxes, one plastic culvert, heavy rubber mats, and a digging area with wood shavings Approximate area: 65 m^2^
Lower Habitat	Half-cement and half-dirt habitat with two plastic culverts, two plastic dens, and heavy rubber mats Approximate area: 45 m^2^

**Table 2:** List of enrichment items given each day of the week and the goal of those items.

Day of the Week	Enrichment Item	Goal of Item
Sunday	Paper towel treat tubes (including avocado, melon, Mazuri^®^ insectivore diet)	Foraging
Monday	Slow feeders with food (including avocado, melon, Mazuri^®^ insectivore diet)	Foraging
Tuesday	Pool with water	Exploring
Wednesday	Containers with crickets	Foraging
Thursday	Warthog-scented towels	Exploring
Friday	Rubber toys with food (including avocado, melon, Mazuri^®^ insectivore diet)	Foraging
Saturday	Perfumed Boomer balls^®^	Exploring

**Table 3:** Ethogram of behaviors. All behaviors were used for instantaneous scan sampling. Asterisks (*) are used to indicate behaviors that were also scored using all-occurrence sampling. For social all-occurrence behaviors, initiators and recipients were noted. Triangles (▲) are used to indicate behaviors that were scored using continuous sampling as well.

Behavior	Description
Affiliative Interaction *	Focal is engaged in any positive social interaction with another individual such as calm contact, sniffing, or social play.
Agonistic Interaction *	Focal is engaged in any negative social interaction with another individual such as displacing, being displaced, biting, hitting with front limbs or torso, being withdrawn from, or withdrawing from an attempt at an affiliative interaction.
Investigate Enrichment ^▲^	Focal is exploring an enrichment item through consuming, touching, smelling, or visual inspection. Focal can be stationary or in motion.
Investigate Object	Focal is exploring a non-food, non-enrichment object or airspace in the habitat through touching, smelling, or visual inspection. Focal can be stationary or in motion.
Pace ^▲^	Focal is in motion but traveling the same route repeatedly. Must have completed the circuit three times before being considered pacing.
Dig	Focal is using anterior or posterior limbs to remove the substrate from a specific area.
Drink	Focal is ingesting water.
Eat	Focal is consuming, touching, smelling, or visually inspecting food items that are not enrichment items.
Groom	Focal’s anterior/posterior limbs are in contact with its own body.
Locomote	Focal is in motion, traveling from one point of the habitat to another, and the head is up off the ground.
Maintenance	Focal is engaged in voiding behaviors.
Stationary–Lay	Focal is inactive with the torso against the ground, but the head is up. Focal may be oriented towards an object/event or just scanning the environment.
Stationary–Stand/Sit	Focal is inactive with the torso off the ground. This behavior includes when a focal is in a seated position. Focal may be oriented towards an object/event or just scanning the environment.
Social Rest	Focal and another aardvark are within one body length of each other, and both are in a state of inactivity with their torsos against the ground and little to no bodily movement. Head is lowered, and eyes may be closed.
Rest	Focal is in a state of inactivity with the torso against the ground; there will be little to no bodily movement. Head is lowered, and eyes may be closed. No other aardvark is resting within one body length of the focal.
Other	Focal is engaged in any behavior not defined elsewhere in the ethogram.
Not Visible	Focal or his/her behavior is out of sight.

## Supplementary Materials

The following are available online at https://www.mdpi.com/2076-2615/10/8/1433/s1, Figure S1: Activity budget (mean ± SE) for the aardvarks (*n* = 4) by stated enrichment goal, Table S1: Pairwise comparisons of counts of scan and continuous event data on enrichment use by stated enrichment goal by the time of night, Table S2: Pairwise comparisons of counts of scan and continuous event data on enrichment use for each time of night by stated enrichment goal, Table S3: Pairwise comparisons of counts of scan and continuous event data on enrichment use for each item by the time of night, Table S4: Pairwise comparisons of counts of scan and continuous event data on enrichment use for the time of night by each item, Table S5: Pairwise comparisons for counts of scan data on the total time foraging by the specific enrichment item presented, Table S6: Pairwise comparisons for counts of scan data on the total time investigating non-food items by the specific enrichment item presented, Table S7: Pairwise comparisons for counts of scan data on the total time locomoting by the specific enrichment item presented, Table S8: Pairwise comparisons for counts of scan and continuous event data on enrichment use based on the interaction between stated enrichment goal and time in the study, Table S9: Pairwise comparisons for counts of scan and continuous event data on enrichment use based on the interaction between enrichment item and time in the study, Table S10: Pairwise comparisons for fecal glucocorticoid metabolite (FGM) concentrations by the specific enrichment item presented, Table S11: Pairwise comparisons for behavioral diversity by the specific enrichment item presented.
