# Supplementary material for: Evaluation of the Impact of Behavioral Opportunities on Four Zoo-Housed Aardvarks (Orycteropus afer)
Source: Animals (Basel). 2020 Aug 17;10(8):1433. doi: 10.3390/ani10081433 (PMC7460251; doi:10.3390/ani10081433)
Supplement: Supplementary file 1 [file animals-10-01433-s001.pdf]

animals

Supplementary Materials: Evaluation of the Impact of Behavioral Opportunities on Four Zoo-Housed Aardvarks (*Orycteropus afer*)

Jennifer Hamilton [1,*], Grace Fuller [1] and Stephanie Allard [2]

[1] Center for Zoo and Aquarium Animal Welfare and Ethics, Detroit Zoological Society, Royal Oak, MI 48067, USA; gfuller@dzs.org
[2] National Aquarium, Baltimore, MD 21202, USA; sallard@aqua.org
* Correspondence: jhamilton@dzs.org; Tel.: +1-248-336-5798

Figure S1. Activity budget (mean ±SE) for the aardvarks ($n = 4$) by stated enrichment goal.

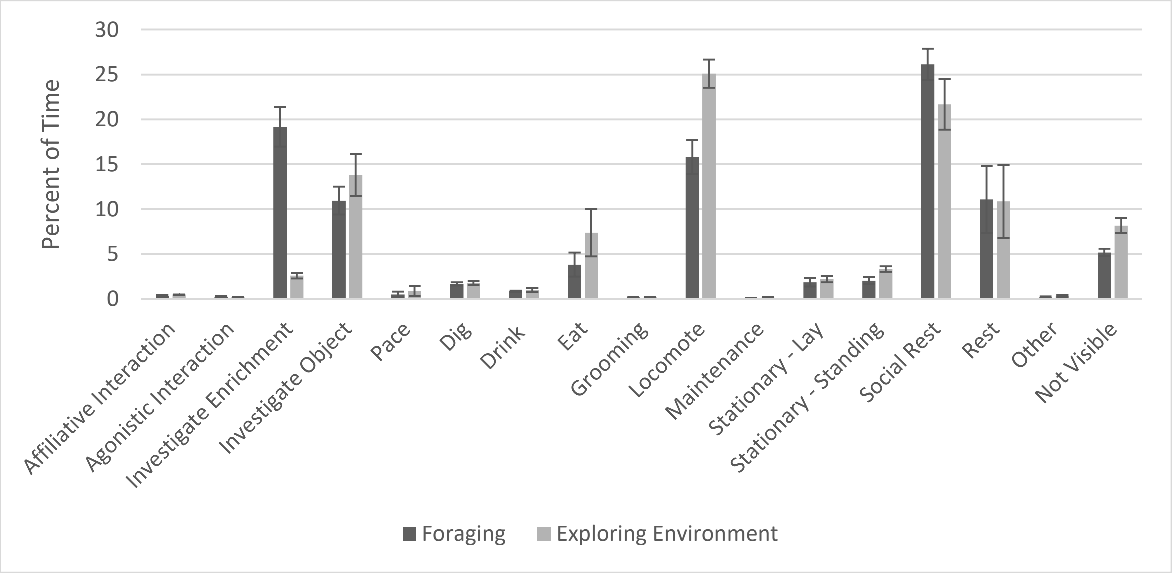

Table S1. Pairwise comparisons of counts of scan and continuous event data on enrichment use by stated enrichment goal by the time of night.

		PM to MID	PM to AM	MID to AM
Foraging Enrichment	Scan Data	$t_{6} = 11.99$, $p < 0.001$	$t_{6} = 12.04$, $p < 0.001$	$t_{664} = 3.39$, $p = 0.001$
	Continuous Event Data	$t_{13} = 8.80$, $p < 0.001$	$t_{12} = 9.10$, $p < 0.001$	$t_{290} = 4.07$, $p < 0.001$
Exploring Environment Enrichment	Scan Data	$t_{231} = 4.85$, $p < 0.001$	$t_{264} = 4.70$, $p < 0.001$	$t_{664} = -0.20$, $p = 0.841$
	Continuous Event Data	$t_{58} = 6.08$, $p < 0.001$	$t_{39} = 6.74$, $p < 0.001$	$t_{664} = 1.77$, $p = 0.076$

Table S2. Pairwise comparisons of counts of scan and continuous event data on enrichment use for each time of night by stated enrichment goal. A graphical representation of the data can be seen in Figure 1.

	**Comparison of Exploring vs. Foraging Enrichment**	
	**Scan Data**	**Continuous Event Data**
PM	$t_{8} = -11.03$, $p < 0.001$	$t_{24} = -5.14$, $p < 0.001$
MID	$t_{151} = 0.03$, $p = 0.975$	$t_{71} = 1.69$, $p = 0.095$
AM	$t_{352} = 3.10$, $p = 0.002$	$t_{125} = 3.45$, $p = 0.001$

Table S3. Pairwise comparisons of counts of scan and continuous event data on enrichment use for each item by the time of night.

		**PM to MID**	**PM to AM**	**MID to AM**
Slow Feeders	Scan Data	$t_{8} = 7.06$, $p < 0.001$	$t_{8} = 7.07$, $p < 0.001$	$t_{649} = 0.53$, $p = 0.594$
	Continuous Event Data	$t_{65} = 6.05$, $p < 0.001$	$t_{55} = 6.30$, $p < 0.001$	$t_{649} = 1.57$, $p = 0.117$
Containers with Crickets	Scan Data	$t_{10} = 6.70$, $p < 0.001$	$t_{10} = 6.74$, $p < 0.001$	$t_{649} = 0.77$, $p = 0.441$
	Continuous Event Data	$t_{57} = 6.24$, $p < 0.001$	$t_{45} = 6.64$, $p < 0.001$	$t_{635} = 3.42$, $p = 0.001$
Rubber Toys with Food	Scan Data	$t_{9} = 6.99$, $p < 0.001$	$t_{8} = 7.07$, $p < 0.001$	$t_{159} = 3.38$, $p = 0.001$
	Continuous Event Data	$t_{53} = 6.35$, $p < 0.001$	$t_{43} = 6.69$, $p < 0.001$	$t_{649} = 3.22$, $p = 0.001$
Paper Towel Treat Tubes	Scan Data	$t_{7} = 7.28$, $p < 0.001$	$t_{7} = 7.29$, $p < 0.001$	$t_{649} = 1.84$, $p = 0.067$
	Continuous Event Data	$t_{56} = 6.28$, $p < 0.001$	$t_{54} = 6.34$, $p < 0.001$	$t_{649} = 0.49$, $p = 0.624$
Pool	Scan Data	$t_{649} = -0.22$, $p = 0.827$	$t_{649} = 1.57$, $p = 0.117$	$t_{649} = 1.76$, $p = 0.079$
	Continuous Event Data	$t_{506} = 3.62$, $p < 0.001$	$t_{207} = 4.53$, $p < 0.001$	$t_{649} = 1.65$, $p = 0.100$
Warthog Scented Towels	Scan Data	$t_{40} = 4.78$, $p < 0.001$	$t_{76} = 4.07$, $p < 0.001$	$t_{649} = -1.51$, $p = 0.130$
	Continuous Event Data	$t_{240} = 4.36$, $p < 0.001$	$t_{131} = 5.08$, $p < 0.001$	$t_{649} = 1.52$, $p = 0.130$
Perfumed Boomer Balls®	Scan Data	$t_{649} = 0.38$, $p = 0.706$	$t_{649} = 1.28$, $p = 0.20$	$t_{649} = 0.95$, $p = 0.342$
	Continuous Event Data	$t_{145} = 4.95$, $p < 0.001$	$t_{157} = 4.84$, $p < 0.001$	$t_{649} = -0.23$, $p = 0.818$

Table S4: Pairwise comparisons of counts of scan and continuous event data on enrichment use for the time of night by each item. Statistics are written for the item on the left side being compared to the item across the top of the table. Continuous event count data is reported below and to the left of the black squares, and scan data is reported to the right and above of the black squares. PM Time Frame right after the enrichment was given (21:00 to 22:00). MID Time Frame (00:30 to 1:30). AM Time Frame (04:00 to 05:00). A graphical representation of the data can be seen in Figure 2.

	**PM**	**Foraging Enrichment**				**Exploring Environment Enrichment**		
		**Slow Feeders**	**Containers with Crickets**	**Rubber Toys with Food**	**Paper Towel Treat Tubes**	**Pool**	**Warthog Scented Towels**	**Perfumed Boomer Balls®**
Foraging Enrichment	Slow Feeders		$t_{20} = 2.84$, $p = 0.010$	$t_{16} = 0.15$, $p = 0.885$	$t_{16} = -2.77$, $p = 0.014$	$t_{9} = 6.97$, $p < 0.001$	$t_{13} = 5.94$, $p < 0.001$	$t_{8} = 7.04$, $p < 0.001$
	Containers with Crickets	$t_{82} = 0.67$, $p = 0.507$		$t_{20} = -2.71$, $p = 0.014$	$t_{16} = -4.79$, $p < 0.001$	$t_{11} = 6.58$, $p < 0.001$	$t_{22} = 4.76$, $p < 0.001$	$t_{10} = 6.71$, $p < 0.001$
	Rubber Toys with Food	$t_{81} = 1.38$, $p = 0.172$	$t_{79} = 0.73$, $p = 0.469$		$t_{16} = -2.89$, $p = 0.011$	$t_{9} = 6.94$, $p < 0.001$	$t_{14} = 5.89$, $p < 0.001$	$t_{9} = 7.02$, $p < 0.001$
	Paper Towel Treat Tubes	$t_{85} = -0.56$, $p = 0.574$	$t_{84} = -1.22$, $p = 0.226$	$t_{82} = -1.91$, $p = 0.060$		$t_{8} = 7.21$, $p < 0.001$	$t_{10} = 6.61$, $p < 0.001$	$t_{7} = 7.26$, $p < 0.001$
Exploring Environment Enrichment	Pool	$t_{75} = -4.96$, $p < 0.001$	$t_{68} = -5.25$, $p < 0.001$	$t_{62} = -5.51$, $p < 0.001$	$t_{83} = -4.70$, $p < 0.001$		$t_{30} = -5.00$, $p < 0.001$	$t_{649} = 1.49$, $p = 0.138$
	Warthog Scented Towels	$t_{89} = -1.33$, $p = 0.187$	$t_{87} = -1.95$, $p = 0.054$	$t_{84} = -2.59$, $p = 0.011$	$t_{90} = -0.78$, $p = 0.438$	$t_{94} = 4.27$, $p < 0.001$		$t_{23} = 5.43$, $p < 0.001$
	Perfumed Boomer Balls®	$t_{87} = -4.17$, $p < 0.001$	$t_{79} = -4.55$, $p < 0.001$	$t_{71} = -4.91$, $p < 0.001$	$t_{94} = -3.82$, $p < 0.001$	$t_{166} = 1.50$, $p = 0.136$	$t_{104} = -3.26$, $p = 0.001$	

**MID**		**Foraging Enrichment**				**Exploring Environment Enrichment**		
		**Slow Feeders**	**Containers with Crickets**	**Rubber Toys with Food**	**Paper Towel Treat Tubes**	**Pool**	**Warthog Scented Towels**	**Perfumed Boomer Balls®**
Foraging Enrichment	Slow Feeders		$t_{649} = -0.20$, $p = 0.844$	$t_{649} = -1.60$, $p = 0.111$	$t_{649} = 0.50$, $p = 0.614$	$t_{649} = -0.60$, $p = 0.548$	$t_{649} = -1.53$, $p = 0.127$	$t_{649} = 1.45$, $p = 0.147$
	Containers with Crickets	$t_{480} = -0.23$, $p = 0.821$		$t_{649} = -1.42$, $p = 0.157$	$t_{649} = 0.70$, $p = 0.484$	$t_{649} = -0.41$, $p = 0.684$	$t_{649} = -1.35$, $p = 0.177$	$t_{649} = 1.63$, $p = 0.103$
	Rubber Toys with Food	$t_{464} = -0.07$, $p = 0.947$	$t_{490} = 0.16$, $p = 0.873$		$t_{589} = 2.04$, $p = 0.042$	$t_{649} = 1.03$, $p = 0.303$	$t_{649} = 0.06$, $p = 0.956$	$t_{283} = 2.80$, $p = 0.005$
	Paper Towel Treat Tubes	$t_{586} = -2.03$, $p = 0.043$	$t_{649} = -1.82$, $p = 0.069$	$t_{610} = -1.97$, $p = 0.050$		$t_{649} = -1.09$, $p = 0.275$	$t_{649} = -1.97$, $p = 0.049$	$t_{649} = 0.98$, $p = 0.326$
Exploring Environment Enrichment	Pool	$t_{556} = -1.12$, $p = 0.265$	$t_{601} = -0.90$, $p = 0.371$	$t_{573} = -1.05$, $p = 0.294$	$t_{649} = 0.98$, $p = 0.330$		$t_{649} = -0.97$, $p = 0.334$	$t_{649} = 1.98$, $p = 0.048$
	Warthog Scented Towels	$t_{216} = 2.90$, $p = 0.004$	$t_{213} = 3.08$, $p = 0.002$	$t_{215} = 2.95$, $p = 0.003$	$t_{158} = 4.28$, $p < 0.001$	$t_{189} = 3.71$, $p < 0.001$		$t_{314} = 2.72$, $p = 0.007$
	Perfumed Boomer Balls®	$t_{584} = -1.92$, $p = 0.056$	$t_{649} = -1.71$, $p = 0.088$	$t_{607} = -1.86$, $p = 0.064$	$t_{649} = 0.13$, $p = 0.900$	$t_{649} = -0.86$, $p = 0.393$	$t_{162} = -4.21$, $p < 0.001$	

	**AM**	**Foraging Enrichment**				**Exploring Environment Enrichment**		
		**Slow Feeders**	**Containers with Crickets**	**Rubber Toys with Food**	**Paper Towel Treat Tubes**	**Pool**	**Warthog Scented Towels**	**Perfumed Boomer Balls®**
Foraging Enrichment	Slow Feeders		$t_{649} = 0.03$, $p = 0.974$	$t_{649} = 1.85$, $p = 0.065$	$t_{649} = 1.82$, $p = 0.069$	$t_{649} = 0.66$, $p = 0.508$	$t_{171} = -3.11$, $p = 0.002$	$t_{649} = 1.79$, $p = 0.074$
	Containers with Crickets	$t_{649} = -2.40$, $p = 0.016$		$t_{649} = 1.84$, $p = 0.066$	$t_{649} = 1.81$, $p = 0.071$	$t_{649} = 0.63$, $p = 0.526$	$t_{168} = -3.13$, $p = 0.002$	$t_{649} = 1.77$, $p = 0.077$
	Rubber Toys with Food	$t_{649} = -2.00$, $p = 0.05$	$t_{649} = 0.50$, $p = 0.620$		$t_{649} = -0.04$, $p = 0.970$	$t_{649} = -1.32$, $p = 0.186$	$t_{75} = -4.06$, $p < 0.001$	$t_{649} = -0.07$, $p = 0.941$
	Paper Towel Treat Tubes	$t_{649} = -1.05$, $p = 0.296$	$t_{649} = 1.49$, $p = 0.137$	$t_{649} = 1.03$, $p = 0.306$		$t_{649} = -1.29$, $p = 0.199$	$t_{76} = -4.05$, $p < 0.001$	$t_{649} = -0.04$, $p = 0.971$
Exploring Environment Enrichment	Pool	$t_{649} = -1.22$, $p = 0.222$	$t_{649} = 1.32$, $p = 0.187$	$t_{649} = 0.85$, $p = 0.396$	$t_{649} = -0.18$, $p = 0.855$		$t_{121} = -3.51$, $p = 0.001$	$t_{649} = 1.25$, $p = 0.212$
	Warthog Scented Towels	$t_{299} = 2.97$, $p = 0.003$	$t_{175} = 4.48$, $p < 0.001$	$t_{194} = 4.26$, $p < 0.001$	$t_{246} = 3.70$, $p < 0.001$	$t_{236} = 3.81$, $p < 0.001$		$t_{76} = 4.04$, $p < 0.001$
	Perfumed Boomer Balls®	$t_{649} = -0.23$, $p = 0.822$	$t_{649} = 2.16$, $p = 0.031$	$t_{649} = 1.76$, $p = 0.079$	$t_{649} = 0.81$, $p = 0.419$	$t_{649} = 0.98$, $p = 0.326$	$t_{296} = -3.13$, $p = 0.002$	

Table S5. Pairwise comparisons for counts of scan data on the total time foraging by the specific enrichment item presented. Statistics are written for the item on the left side being compared to the item across the top of the table. A graphical representation of the data can be seen in Figure 3.

		**Foraging Enrichment**				**Exploring Environment Enrichment**		
		**Slow Feeders**	**Containers with Crickets**	**Rubber Toys with Food**	**Paper Towel Treat Tubes**	**Pool**	**Warthog Scented Towels**	**Perfumed Boomer Balls®**
Foraging Enrichment	Slow Feeders							
	Containers with Crickets	$t_{34} = -1.20$, $p = 0.239$						
	Rubber Toys with Food	$t_{31} = 0.117$, $p = 0.908$	$t_{34} = 1.30$, $p = 0.201$					
	Paper Towel Treat Tubes	$t_{32} = 1.48$, $p = 0.150$	$t_{30} = 2.45$, $p = 0.020$	$t_{31} = 1.37$, $p = 0.180$				
Exploring Environment Enrichment	Pool	$t_{12} = -4.26$, $p = 0.001$	$t_{15} = -3.95$, $p = 0.001$	$t_{12} = -4.28$, $p = 0.001$	$t_{10} = -4.51$, $p = 0.001$			
	Warthog Scented Towels	$t_{19} = -3.61$, $p = 0.002$	$t_{27} = -3.04$, $p = 0.005$	$t_{18} = -3.65$, $p = 0.002$	$t_{13} = -4.09$, $p = 0.001$	$t_{49} = 2.08$, $p = 0.043$		
	Perfumed Boomer Balls®	$t_{13} = -4.11$, $p = 0.001$	$t_{17} = -3.75$, $p = 0.002$	$t_{13} = -4.14$, $p = 0.001$	$t_{11} = -4.42$, $p = 0.001$	$t_{61} = 0.64$, $p = 0.527$	$t_{51} = -1.55$, $p = 0.128$	

Table S6. Pairwise comparisons for counts of scan data on the total time investigating non-food items by the specific enrichment item presented. Statistics are written for the item on the left side being compared to the item across the top of the table. A graphical representation of the data can be seen in Figure 4.

		**Foraging Enrichment**				**Exploring Environment Enrichment**		
		**Slow Feeders**	**Containers with Crickets**	**Rubber Toys with Food**	**Paper Towel Treat Tubes**	**Pool**	**Warthog Scented Towels**	**Perfumed Boomer Balls®**
Foraging Enrichment	Slow Feeders							
	Containers with Crickets	$t_{48} = -0.15$, $p = 0.883$						
	Rubber Toys with Food	$t_{49} = -0.55$, $p = 0.582$	$t_{49} = -0.41$, $p = 0.686$					
	Paper Towel Treat Tubes	$t_{50} = -0.76$, $p = 0.450$	$t_{50} = -0.617$, $p = 0.540$	$t_{51} = -0.21$, $p = 0.833$				
Exploring Environment Enrichment	Pool	$t_{46} = 1.56$, $p = 0.126$	$t_{46} = 1.69$, $p = 0.098$	$t_{46} = 2.032$, $p = 0.048$	$t_{46} = 2.20$, $p = 0.033$			
	Warthog Scented Towels	$t_{42} = 2.39$, $p = 0.021$	$t_{41} = 2.50$, $p = 0.017$	$t_{38} = 2.78$, $p = 0.008$	$t_{36} = 2.92$, $p = 0.006$	$t_{41} = 1.00$, $p = 0.325$		
	Perfumed Boomer Balls®	$t_{46} = 0.89$, $p = 0.378$	$t_{47} = 1.03$, $p = 0.307$	$t_{49} = 1.41$, $p = 0.164$	$t_{49} = 1.60$, $p = 0.115$	$t_{43} = -0.71$, $p = 0.482$	$t_{43} = -1.65$, $p = 0.105$	

Table S7: Pairwise comparisons for counts of scan data on the total time locomoting by the specific enrichment item presented. Statistics are written for the item on the left side being compared to the item across the top of the table. A graphical representation of the data can be seen in Figure 5.

		Foraging Enrichment				Exploring Environment Enrichment		
		**Slow Feeders**	**Containers with Crickets**	**Rubber Toys with Food**	**Paper Towel Treat Tubes**	**Pool**	**Warthog Scented Towels**	**Perfumed Boomer Balls®**
Foraging Enrichment	Slow Feeders							
	Containers with Crickets	$t_{47} = -0.212$, $p = 0.833$						
	Rubber Toys with Food	$t_{49} = -1.04$, $p = 0.304$	$t_{49} = -0.834$, $p = 0.409$					
	Paper Towel Treat Tubes	$t_{49} = -1.32$, $p = 0.192$	$t_{49} = -1.12$, $p = 0.268$	$t_{50} = -0.30$, $p = 0.769$				
Exploring Environment Enrichment	Pool	$t_{46} = 2.24$, $p = 0.030$	$t_{46} = 2.42$, $p = 0.020$	$t_{46} = 3.06$, $p = 0.004$	$t_{46} = 3.26$, $p = 0.002$			
	Warthog Scented Towels	$t_{46} = 1.29$, $p = 0.203$	$t_{46} = 1.49$, $p = 0.143$	$t_{48} = 2.23$, $p = 0.030$	$t_{48} = 2.48$, $p = 0.017$	$t_{43} = -1.05$, $p = 0.298$		
	Perfumed Boomer Balls®	$t_{46} = 0.92$, $p = 0.361$	$t_{46} = 1.13$, $p = 0.266$	$t_{48} = 1.90$, $p = 0.063$	$t_{49} = 2.16$, $p = 0.036$	$t_{44} = -1.42$, $p = 0.163$	$t_{44} = -0.38$, $p = 0.703$	

Table S8. Pairwise comparisons for counts of scan and continuous event data on enrichment use based on the interaction between stated enrichment goal and time in the study.

		Weeks 1-2 to Weeks 3-4	Weeks 1-2 to Weeks 5-6	Weeks 1-2 to Weeks 7-8	Weeks 3-4 to Weeks 5-6	Weeks 3-4 to Weeks 7-8	Weeks 5-6 to Weeks 7-8
Foraging Enrichment	Scan Data	$t_{216} = -1.20$, $p = 0.230$	$t_{216} = -0.51$, $p = 0.608$	$t_{216} = -1.16$, $p = 0.248$	$t_{216} = 0.70$, $p = 0.486$	$t_{216} = 0.05$, $p = 0.962$	$t_{216} = -0.65$, $p = 0.516$
	Continuous Event Data	$t_{216} = -0.76$, $p = 0.448$	$t_{216} = 0.36$, $p = 0.720$	$t_{216} = 0.66$, $p = 0.510$	$t_{216} = 1.12$, $p = 0.265$	$t_{216} = 1.41$, $p = 0.159$	$t_{216} = 0.30$, $p = 0.763$
Exploring Environment Enrichment	Scan Data	$t_{216} = -1.38$, $p = 0.169$	$t_{216} = 0.59$, $p = 0.553$	$t_{216} = -0.58$, $p = 0.564$	$t_{216} = 1.94$, $p = 0.054$	$t_{216} = 0.82$, $p = 0.413$	$t_{216} = -1.16$, $p = 0.247$
	Continuous Event Data	$t_{216} = -0.45$, $p = 0.656$	$t_{216} = 1.67$, $p = 0.096$	$t_{216} = 0.43$, $p = 0.669$	$t_{216} = 2.09$, $p = 0.038$	$t_{216} = 0.87$, $p = 0.384$	$t_{216} = -1.26$, $p = 0.211$

Table S9. Pairwise comparisons for counts of scan and continuous event data on enrichment use based on the interaction between enrichment item and time in the study. A graphical representation of the data can be seen in Figure 6.

		Weeks 1-2 to Weeks 3-4	Weeks 1-2 to Weeks 5-6	Weeks 1-2 to Weeks 7-8	Weeks 3-4 to Weeks 5-6	Weeks 3-4 to Weeks 7-8	Weeks 5-6 to Weeks 7-8
Slow Feeders	Scan Data	$t_{41} = -4.73$, $p < 0.001$	$t_{196} = -3.06$, $p = 0.003$	$t_{35} = -4.93$, $p < 0.001$	$t_{196} = 2.64$, $p = 0.009$	$t_{196} = -0.45$, $p = 0.654$	$t_{196} = -3.00$, $p = 0.003$
	Continuous Event Data	$t_{196} = 0.90$, $p = 0.369$	$t_{196} = 2.98$, $p = 0.003$	$t_{196} = 3.29$, $p = 0.001$	$t_{196} = 2.19$, $p = 0.030$	$t_{196} = 2.53$, $p = 0.012$	$t_{196} = 0.38$, $p = 0.704$
Containers with Crickets	Scan Data	$t_{196} = 0.97$, $p = 0.333$	$t_{196} = -1.38$, $p = 0.171$	$t_{196} = 0.10$, $p = 0.924$	$t_{196} = -2.26$, $p = 0.025$	$t_{196} = -0.88$, $p = 0.381$	$t_{196} = 1.46$, $p = 0.145$
	Continuous Event Data	$t_{196} = -1.46$, $p = 0.145$	$t_{196} = -1.96$, $p = 0.051$	$t_{196} = -1.85$, $p = 0.065$	$t_{196} = -0.53$, $p = 0.600$	$t_{196} = -0.41$, $p = 0.682$	$t_{196} = 0.12$, $p = 0.908$
Rubber Toys with Food	Scan Data	$t_{196} = 0.99$, $p = 0.322$	$t_{196} = 1.65$, $p = 0.102$	$t_{196} = 0.99$, $p = 0.322$	$t_{196} = 0.68$, $p = 0.496$	$t_{196} = -0.001$, $p = 0.999$	$t_{196} = -0.68$, $p = 0.495$
	Continuous Event Data	$t_{196} = -1.00$, $p = 0.317$	$t_{196} = -1.03$, $p = 0.305$	$t_{196} = 0.67$, $p = 0.506$	$t_{196} = -0.03$, $p = 0.979$	$t_{196} = 1.65$, $p = 0.101$	$t_{196} = 1.67$, $p = 0.096$

Paper Towel Treat Tubes	Scan Data	$t_{196} = -0.97$, $p = 0.333$	$t_{196} = -0.25$, $p = 0.804$	$t_{196} = -0.19$, $p = 0.852$	$t_{196} = 0.73$, $p =$ 0.468	$t_{196} = 0.79$, $p = 0.431$	$t_{196} = 0.06$, $p = 0.950$
	Continuous Event Data	$t_{196} = -0.79$, $p = 0.433$	$t_{196} = 1.16$, $p = 0.246$	$t_{196} = -0.67$, $p = 0.505$	$t_{196} = 1.91$, $p =$ 0.057	$t_{196} = 0.12$, $p = 0.905$	$t_{196} = -1.80$, $p = 0.073$
Pool	Scan Data	$t_{196} = 0.38$, $p = 0.706$	$t_{196} = -0.90$, $p = 0.370$	$t_{196} = 1.76$, $p = 0.081$	$t_{196} = -1.25$, $p =$ 0.214	$t_{196} = -2.06$, $p = 0.041$	$t_{196} = -0.94$, $p = 0.351$
	Continuous Event Data	$t_{196} = -2.18$, $p = 0.030$	$t_{196} = 0.15$, $p = 0.882$	$t_{196} = -0.54$, $p = 0.588$	$t_{196} = 2.31$, $p =$ 0.022	$t_{196} = 1.69$, $p = 0.093$	$t_{196} = -0.69$, $p = 0.492$
Warthog Scented Towels	Scan Data	$t_{196} = -1.83$, $p = 0.069$	$t_{196} = 1.29$, $p = 0.197$	$t_{196} = 0.41$, $p = 0.681$	$t_{196} = 2.91$, $p =$ 0.004	$t_{196} = 2.19$, $p = 0.030$	$t_{196} = -0.90$, $p = 0.370$
	Continuous Event Data	$t_{196} = -0.34$, $p = 0.733$	$t_{196} = 3.09$, $p = 0.002$	$t_{196} = 0.88$, $p = 0.381$	$t_{196} = 3.36$, $p =$ 0.001	$t_{196} = 1.21$, $p = 0.227$	$t_{196} = -2.33$, $p = 0.021$
Perfumed Boomer Balls®	Scan Data	$t_{196} = -0.58$, $p = 0.565$	$t_{196} = -0.58$, $p = 0.565$	$t_{196} = -0.99$, $p = 0.323$	$t_{196} = -7.80E\text{-}06$, $p = 1.00$	$t_{196} = -0.45$, $p = 0.656$	$t_{196} = 0.45$, $p = 0.656$
	Continuous Event Data	$t_{196} = 1.70$, $p = 0.090$	$t_{196} = -1.01$, $p = 0.313$	$t_{196} = 0.01$, $p = 0.989$	$t_{196} = -2.62$, $p =$ 0.010	$t_{196} = -1.69$, $p = 0.092$	$t_{196} = 1.03$, $p = 0.307$

Table S10. Pairwise comparisons for fecal glucocorticoid metabolite (FGM) concentrations by the specific enrichment item presented. There were not enough samples on days that containers with crickets were given to allow for statistical analysis. For this reason, containers with crickets are not shown in the table. Statistics are written for the item on the left side being compared to the item across the top of the table.

		**Foraging Enrichment**			**Exploring Environment Enrichment**		
		**Slow Feeders**	**Rubber Toys with Food**	**Paper Towel Treat Tubes**	**Pool**	**Warthog Scented Towels**	**Perfumed Boomer Balls®**
Foraging Enrichment	Slow Feeders						
	Rubber Toys with Food	$t_{31} = -0.06$, $p = 0.952$					
	Paper Towel Treat Tubes	$t_{42} = 0.51$, $p = 0.613$	$t_{37} = 0.56$, $p = 0.576$				
Exploring Environment Enrichment	Pool	$t_{207} = -0.49$, $p = 0.624$	$t_{207} = 0.91$, $p = 0.364$	$t_{207} = 0.01$, $p = 0.995$			
	Warthog Scented Towels	$t_{49} = -0.26$, $p = 0.800$	$t_{39} = -0.22$, $p = 0.828$	$t_{64} = -0.64$, $p = 0.525$	$t_{53} = 0.97$, $p = 0.335$		
	Perfumed Boomer Balls®	$t_{71} = -1.12$, $p = 0.267$	$t_{63} = -1.12$, $p = 0.265$	$t_{81} = -1.39$, $p = 0.167$	$t_{45} = -0.04$, $p = 0.972$	$t_{36} = -1.15$, $p = 0.260$	

Table S11. Pairwise comparisons for behavioral diversity by the specific enrichment item presented. Statistics are written for the item on the left side being compared to the item across the top of the table.

		**Foraging Enrichment**				**Exploring Environment Enrichment**		
		**Slow Feeders**	**Containers with Crickets**	**Rubber Toys with Food**	**Paper Towel Treat Tubes**	**Pool**	**Warthog Scented Towels**	**Perfumed Boomer Balls®**
Foraging Enrichment	Slow Feeders							
	Containers with Crickets	$t_{207} = 1.03$, $p = 0.303$						
	Rubber Toys with Food	$t_{207} = -1.40$, $p = 0.163$	$t_{207} = -2.43$, $p = 0.016$					
	Paper Towel Treat Tubes	$t_{207} = -0.50$, $p = 0.619$	$t_{207} = -1.53$, $p = 0.128$	$t_{207} = 0.90$, $p = 0.367$				
Exploring Environment Enrichment	Pool	$t_{207} = -0.49$, $p = 0.624$	$t_{207} = -1.52$, $p = 0.129$	$t_{207} = 0.91$, $p = 0.364$	$t_{207} = 0.01$, $p = 0.995$			
	Warthog Scented Towels	$t_{207} = 1.50$, $p = 0.136$	$t_{207} = 0.46$, $p = 0.643$	$t_{207} = 2.90$, $p = 0.004$	$t_{207} = 1.99$, $p = 0.048$	$t_{207} = 1.99$, $p = 0.048$		
	Perfumed Boomer Balls®	$t_{207} = -1.53$, $p = 0.127$	$t_{207} = -2.56$, $p = 0.011$	$t_{207} = -0.13$, $p = 0.896$	$t_{207} = -1.03$, $p = 0.302$	$t_{207} = -1.04$, $p = 0.299$	$t_{207} = -3.03$, $p = 0.003$	
